# Silencing the Monitor That Cried Wolf: A Role for Artificial Intelligence

**DOI:** 10.1016/j.jacadv.2025.101997

**Published:** 2025-07-18

**Authors:** Rohan G. Reddy, David A. Danford, Shelby Kutty

**Affiliations:** a Department of Biology, The Johns Hopkins University, Baltimore, Maryland, USA; b Academic Programs, BayCare Health System, Clearwater, Florida, USA

The benefits of remote patient monitoring include detection of threatening conditions and reduction of the physical and financial burden of frequent office visits. Implantable cardiac monitors (ICMs) provide many of the benefits of remote patient monitoring, but at the price of a large number of insignificant or nonactionable alerts (NAAs) that clinicians must review. ICMs enhanced by artificial intelligence (AI) are relatively new but already show potential to reduce time and resources consumed in the process of monitoring ICM alerts.

We therefore congratulate Katapadi et al^1^ for their fine paper evaluating the impact of AI-enhanced ICMs on device clinic workflow and resource utilization. Their AI-enhanced algorithm was rigorously trained and validated on independent datasets, where it exhibited nearly perfect accuracy. It boosted efficiency by reducing the total number of NAAs, thereby reducing associated staffing time and costs.

Some may worry that adoption of AI-enhanced ICMs might be slowed by the algorithm opacity that they share with many other risk stratification “black box” AI models.^2^ Unfortunately, the alternative in which NAAs are flagged and still presented to the physician would largely sacrifice the advantages reported in this paper. One potential benefit that could not be addressed by this study was whether clinician reviewer performance might be enhanced by using AI to reduce alarm fatigue.^3^ In other words, would reviewers classify recordings correctly more often when they are relieved of the flood of NAAs they would otherwise be receiving? It is time for a prospective evaluation of the performance of clinician reviewers as they receive recordings presented to them by AI-enhanced ICMs in actual practice. We predict that, were we to discover that alarm fatigue is reduced and reviewer performance is superior, even a relatively opaque algorithm will be eagerly embraced.
